# Ground beef microbiome changes with antimicrobial decontamination interventions and product storage

**DOI:** 10.1371/journal.pone.0217947

**Published:** 2019-06-05

**Authors:** Margaret D. Weinroth, Brianna C. Britton, Kathryn R. McCullough, Jennifer N. Martin, Ifigenia Geornaras, Rob Knight, Keith E. Belk, Jessica L. Metcalf

**Affiliations:** 1 Department of Animal Sciences, Colorado State University, Fort Collins, Colorado, United States of America; 2 Department of Food Science, Purdue University, West Lafayette, Indiana, United States of America; 3 North American Meat Institute, Washington D.C., United States of America; 4 Departments of Pediatrics, Bioengineering, and Computer Science and Engineering, and Center for Microbiome Innovation, University of California San Diego, La Jolla, California, United States of America; South Dakota State University, UNITED STATES

## Abstract

Ground beef makes up more than half of the beef consumed in the U.S. market. Although numerous studies have been conducted on microbial safety and shelf life of ground beef limited work has been done using a culture-independent approach. While past studies have allowed for the evaluation of a few organisms of interest, there is limited work on the microbial community associated with fresh ground beef. In order to have a more complete picture of the microbial ecology of the product, a culture-independent approach utilizing 16S rRNA gene amplicon sequencing was used. The objectives of this study were to characterize the fresh ground beef microbiome and the effect that antimicrobial interventions and antioxidants, applied to beef trim before grinding, and product storage have on community composition using 16S rRNA gene amplicon sequencing. Beef trimmings were treated with antimicrobials and an antioxidant. Samples were ground, loafed, and overwrapped before being packaged in modified-atmosphere packaging. Samples were in dark storage for 21 days followed by five days in retail display. Periodically during storage, samples were collected for microbiological analysis and DNA isolation. Due to low microbial biomass, only 52 of 210 samples were included in the final analysis. These samples represented two antimicrobial treatments (peroxyacetic acid, and a sulfuric acid and sodium sulfate blend) and a control, from day-15 of dark storage and day-5 of retail display. As sample age increased, so did the number of raw reads (*P* < 0.001) and aerobic plate counts (*P* < 0.001), which were correlated (*r* = 0.94, *P* = 0.017). Across all samples, lactic acid bacteria were most abundant followed by *Enterobacteriaceae*; several rare taxa were also identified (namely *Geobacillus*, *Thermus*, and *Sporosarcina*). Antimicrobial treatment altered the bacterial alpha (*P* < 0.001) and beta (*P* = 0.001) diversity, while storage day altered alpha (*P =* 0.001) diversity. *Enterobacteriaceae* relative abundance differed (*P* < 0.05) among treatments and was highest in control samples. In addition to confirming previously described dominant microbial differences in culture-dependent results, these data identified genera not typically associated with ground beef and allowed for study of shifts in the entire microbiome and not just a subset of indicator organisms.

## Introduction

Beef is a widely consumed protein in the U.S. market, with an estimated per capita consumption of 54.4 lbs in 2017 [1]. Of this, an estimated 62% of all beef sold is ground [2]. The marketing of ground beef relies on a safe product that has an acceptable shelf life. In terms of safety, *Escherichia coli* O157:H7, non-O157 Shiga toxin-producing *E*. *coli* (STEC), and *Salmonella* spp. are pathogens that have been associated with ground beef [3–5]. In conjunction with safety concerns, the quality of ground beef over time has also been extensively studied. While spoilage bacteria (such as *Lactobacillus* spp., *Leuconostoc* spp., and *Pseudomonas* spp. [6]) are not a threat to consumer health, their growth during product storage can decrease shelf life and increase off flavor and odors. Factors such as storage temperature, food safety interventions, packaging type, and length of distribution and retail display all have an effect on the shelf life of the product.

*E*. *coli* O157:H7 and non-O157 STECs are adulterants in ground beef according to the U.S. Department of Agriculture Food Safety and Inspection Service (USDA-FSIS), meaning there is zero-tolerance for their presence in the product [7]. On the other hand, *Salmonella* spp. is governed by a performance standard which sets a maximum threshold allowed in ground beef [8]. The prevalence of these pathogen groups can be reduced through the use of chemical and/or physical decontamination interventions during the slaughter process [9]. Chemical treatments of the meat, such as lactic acid, peroxyacetic acid, and a commercial blend of sulfuric acid and sodium sulfate, have been shown to reduce pathogen contamination on beef products through various methods of application at different stages of processing [10–13].

The shelf life of ground beef can also be extended with the use of chemical interventions, because these interventions do not only target pathogenic bacteria but those involved in spoilage as well. However, although chemical interventions may reduce spoilage bacteria numbers, these interventions may also impact quality attributes that greatly affect consumer preference, such as color [14]. As a result, there must be a balance to the number and type of interventions used that maximizes both safety and quality. In addition to chemical interventions, antioxidants, sometimes used in tandem with chemical interventions, reduce oxidation of lipids and proteins, increasing shelf life [15]. The effect of these interventions on the fresh meat microbiome is unknown.

Another component of the safety and quality of ground beef is packaging. Modified atmosphere packaging (MAP) is a system of packaging that alters the gaseous components of a package allowing for the extended shelf life of a product [16]. Components of MAP packaging often include carbon monoxide (CO), carbon dioxide (CO_2_), and oxygen at different levels. These are combined to maintain color throughout storage, while minimizing microbial growth and lipid oxidation [14]. In addition to increasing shelf life, MAP packaging inhibits pathogen growth, including *E*. *coli* O157:H7 [17] and spoilage organisms [18]. In low oxygen mixtures, these changes in atmosphere likely affect the microbial community associated with fresh meat by selecting for anaerobes or facultative anaerobes.

The efficacy of interventions and packaging technologies in reducing microbial numbers and inhibiting growth during product storage, respectively, has been primarily studied using traditional culture-based methods. While this approach is useful in understanding the changes to a targeted organism of interest, culturing approaches do not capture changes in the unculturable component of the microbiome. Characterizing the full diversity of the bacterial community can provide insights into ecologies that may support or not support the organism of interest, and how the microbial community changes with the application of antimicrobial interventions and subsequent product storage. In order to evaluate these bacterial community changes, sequencing of the 16S rRNA gene can be used to quantify a relative abundance of bacterial taxa present. In terms of the fresh beef microbiome, limited work has been done using culture-independent methods [19,20]. For example, work has been done on ground beef specific to 16S rRNA gene sequencing of cultured lactic acid bacteria isolates [20], while another 16S rRNA gene sequencing study was conducted on whole muscle beef cuts rather than ground product [19]. With this in mind, the objectives of this study were to characterize the fresh ground beef microbiome and the effect of antimicrobial interventions, antioxidants, and storage on this community using 16S rRNA gene amplicon sequencing.

## Materials and methods

### Sample collection and antimicrobial treatment

Ground beef trimmings (80% lean) were collected from a commercial harvest facility, transported to Colorado State University (Fort Collins, CO) within 1 h of collection, and stored overnight at 4°C. The next day, the trim was divided into five different 90 kg batches for subsequent antimicrobial treatment using a custom-built spray cabinet (Birko/Chad Equipment, Olathe, KS) designed for trim and sub-primal cuts. The cabinet was fitted with 18 floodjet spray nozzles (0.38 liter/min; Grainger Industrial Supplies, Lake Forest, IL), with 10 nozzles affixed above the product belt and eight nozzles below. Antimicrobial treatments selected for inclusion in the study are used by the beef industry and their antimicrobial effects against pathogen contamination on beef products has previously been reported [10, 12]. The antimicrobial treatments evaluated were: (1) untreated control, (2) lactic acid (4%, LA; Corbion, Lenexa, KS), (3) a commercially available blend of sulfuric acid and sodium sulfate (pH 1.2, SASS; Zoetis, Parsippany, NJ), (4) peroxyacetic acid (350 ppm, PAA; Kroff Food Service, Inc., Pittsburgh, PA), and (5) PAA (350 ppm) acidified with SASS (pH 1.2, PAA/SASS). Spray treatments were applied at a pressure of 0.15 MPa with a product contact time of 10 to 11 s. Untreated (control) and treated batches of trim were then stored at 4°C for 24 h prior to further processing.

### Grinding, antioxidant application, storage, and display

Trim treatments were separately course ground to 12.7 mm, and after the course grind, each treatment was divided in half. Forty-five kilograms of each treatment was retained for antioxidant application while the other half was fine ground to 3.2 mm. For antioxidant application, 224.2 g (0.494% of the total weight) of a commercially prepared antioxidant (dried vinegar and natural flavors, Wenda Ingredients, Prosur, Murica, Spain) at a 0.3% concentration was added following the initial course grind and mixed for 30 s using a countertop mixer (KitchenAid, Benton Harbor, MI), followed by a fine grind to 3.2 mm. Immediately after grinding, a sample from each antimicrobial/antioxidant combination was retained for microbiological analysis and sequencing. All product was loafed by hand into 450 g loaves and placed onto 2P trays (Team Packaging Inc., Denver, CO) with an absorbent pad and individually overwrapped with a gas permeable plastic film.

Batches of five trays of ground beef of the same treatment were placed into individual 50 × 50 cm modified atmosphere packaging (MAP) bags (SealedAir, Denver, CO), with an oxygen scavenger. The bags were sealed using a MAP packager (Corr-Vac; M-Tek, Elgin, IL) to remove the oxygen and fill the bags with a Tri-Gas mixture (19.6% CO_2_, 0.4% CO, and balancing N; Airgas, Salt Lake City, UT) prior to sealing. Bags were then placed in opaque cardboard boxes, sealed, and stacked in 4°C for up to 21 days in dark storage to emulate common industry practices for storage times. Following the 21-day storage period, the trays of ground beef were removed from the MAP system and were placed in a simulated retail display case (4°C) for up to five days (once again to emulate common industry practices). Samples were collected for microbiological analysis and sequencing on days 0, 6, and 15 of dark storage, and on day 5 of retail display after 21 days of dark storage. It should be noted that additional samples were placed into dark storage and retail display for another study not described in the present work, thus the surplus of ground beef loaves.

### Microbiological analysis

At each sampling time, 10 samples from each of the 10 ground beef treatments (i.e., five antimicrobial treatments, with and without the addition of the antioxidant) were analyzed for aerobic plate counts (APC). For the analysis, a 50-g portion of each sample was aseptically transferred to a filter Whirl-Pak bag, to which 100 mL of Dey-Engley neutralizing broth (Difco, Becton Dickinson [BD], Sparks, MD) was added. Samples were mechanically pummeled for 2 min and then serially-diluted in 0.1% buffered peptone water (Difco, BD). Appropriate dilutions were plated, in duplicate, onto Petrifilm Aerobic Plate Count plates (3M, St. Paul, MN) and colonies were counted after incubation of plates at 35°C for 48 h.

### 16S rRNA gene amplicon sequencing

Dry sterile swabs (Becton Dickinson and Co., Sparks, MD) were used to collect DNA from the same ground beef samples that were analyzed for APC (in addition to a sample immediately after fine grinding). Sampling was performed by inserting the swab into the geometric center of the loaf and rotating the swab several times. After sampling, swabs were returned to their original holder and immediately placed at -20°C before transportation to the University of Colorado-Boulder for DNA extraction. DNA was extracted and processed 1 to 2 months post collection. DNA extraction and sequencing library preparation followed the Earth Microbiome Project standard protocols [21]. Briefly, DNA was extracted from swabs using the MoBio Powersoil 96-well kit (Mo Bio Laboratories, Inc., Carlsbad, California) with the 515F-Y with barcode/806R primer set for amplification of the V4 region [21–23]. 16S rRNA amplicons were sequenced on an Illumina MiSeq at the University of California-San Diego, Institute for Genomic Medicine (150x1).

### Bioinformatics and statistics

Amplicon sequences were processed through the QIIME2 (2018.6) pipeline [24]. Samples were demultiplexed and assigned exact sequence variance (ESV) using the DADA2 plugin [25] with no truncation of the reads and the chimera method set to pooled. Multiple sequence alignment of the sequences was completed with MAFFT [26] and filtered to remove highly variable positions. FastTree 2 [27] was used to construct and root a phylogenetic tree. Taxonomic classification was conducted using a pretrained Naive Bayes classifier trained on the Greengenes database for the 16S rRNA region spanning 515/806 region [28]. Reads assigned to mitochondria and chloroplast were removed from downstream analysis. Samples were rarified at a depth of 8021, allowing retention of 52 samples. This rarefication depth was chosen to maintain as many samples as possible while retaining as much sequence depth as possible; there was a decrease in sequencing depth to 3506 after the sample containing 8021.

Alpha diversity was assessed via Faith’s Phylogenetic Diversity and beta diversity was measured using weighted UniFrac distances (unweighted results found in S1 Fig). Alpha diversity, after verification of a normal distribution using the ‘shapiro.test’ function, was compared using the lm and anova functions in R version 3.5.1 and compared using the CLD function from the emmeans package. Beta diversity interactions and main effects were evaluated using the adonis function from Vegan (v. 2.5–2). Differential abundance was conducted at the aggregated family level via ANCOM [29]. In all comparisons, *α* = 0.05 and an FDR adjustment was used when appropriate.

Colony counts recovered from the Petrifilm Aerobic Count plates were converted to log CFU/g. While there was an interaction present between sampling day and treatment, as determined by the ‘glm’ and ‘emmeans’ functions in base R (v. 3.5.1) and emmeans package (v. 1.2.3), the treatments were pooled across day for the comparison of sequenced reads to APC. The APCs were averaged to obtain an APC per day of the study, and the same was done for raw sequences. The ‘cor.test’ function was used with default settings to determine if there was a correlation between number of reads sequenced and APC by sampling day.

### Accession number

Samples described in this study have been deposited on QIITA, ID 10937 and EBI ERP113446.

## Results and discussion

### General sequencing results

Sequencing of the 16S rRNA V4 partial gene region of 210 DNA samples isolated from swabs of ground beef (and 31 blank swabs) generated 1.6M reads with an average of 7391 reads per sample (range 0 to 53708). Quality filtering resulted in removal of 1.5% of reads across all samples. Of remaining filtered reads, 0.96% were classified as chimeric and removed. Sequencing depth was considered appropriate via the construction of a rarefaction curve (S2 Fig). Due to the low biomass of bacteria and subsequent low number of sequenced reads found within specific storage days and antimicrobial treatments, only 52 samples were included for downstream analysis (Table 1). Of the blank sample swabs, 11 sequenced with an average read count of only 12 reads.

**Table 1:** Details of the 52 samples included in the analysis that had enough biomass for downstream analysis.

Storage Day	Antimicrobial Treatment Applied to Beef Trim			Total
	SASS^1^	None	PAA^2^	
Day-15 of dark storage	11	10	8	29
Day-5 of retail display^3^	8	8	7	23
**Total**	**19**	**18**	**15**	**52**

^1^ Sulfuric acid and sodium sulfate blend

^2^ Peroxyacetic acid

^3^ Retail display followed 21 days of dark storage

### Culture results agree with low biomass sequencing results

**Fig 1:**
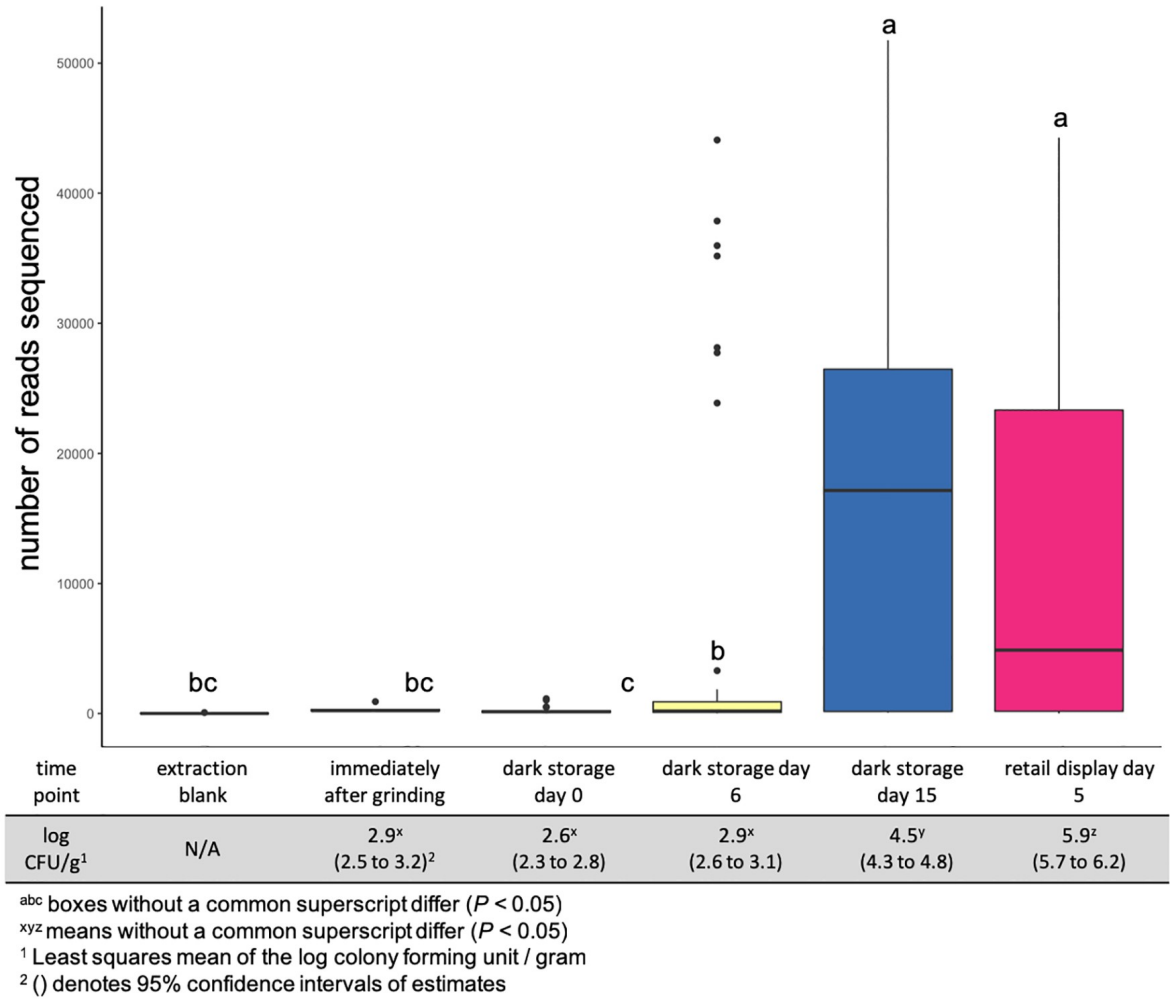
Raw reads sequenced versus aerobic plate counts by day. Number of raw reads sequenced per storage day of the study compared to aerobic plate counts (APC) recovered from the same samples on the same day. There was an increase (*P* < 0.05) in both raw reads and APC populations over the duration of the study. These two measurements were found to be correlated to each other (*r* = 0.94, *P* = 0.017).

Ground beef sampled at later times during the study generated a greater (*P* > 0.001) number of reads per sample (Fig 1), regardless of antimicrobial treatment. Aerobic plate counts (APC) also increased (*P* > 0.001) by day of sampling (Fig 1). In fact, the number of read sequences and APC counts were highly correlated (*r* = 0.94, *P* = 0.017). The increase of culturable bacteria during retail display has been previously documented. Brooks et al. [30] observed an increase in psychrophilic APC in low-oxygen MAP packaging over a 21 day retail display period while Lavieri and Williams [31] reported a similar trend in regards to ground beef during storage. The correlation between the culture-independent and culture approach demonstrates that early on in ground beef storage, the microbial community biomass is very low.

### Antioxidants had no effect on the ground beef microbiome

In this study, some of the antimicrobial interventions had antioxidants added (S1 Table). However, it was found there was no difference in alpha (*P* = 0.79) or beta diversity (*P* = 0.38) between ground beef microbiomes treated and not treated with the antioxidants. As a result, this variable was not considered in downstream analysis.

### Firmicutes dominated the microbiome, regardless of storage day or antimicrobial treatment

**Fig 2:**
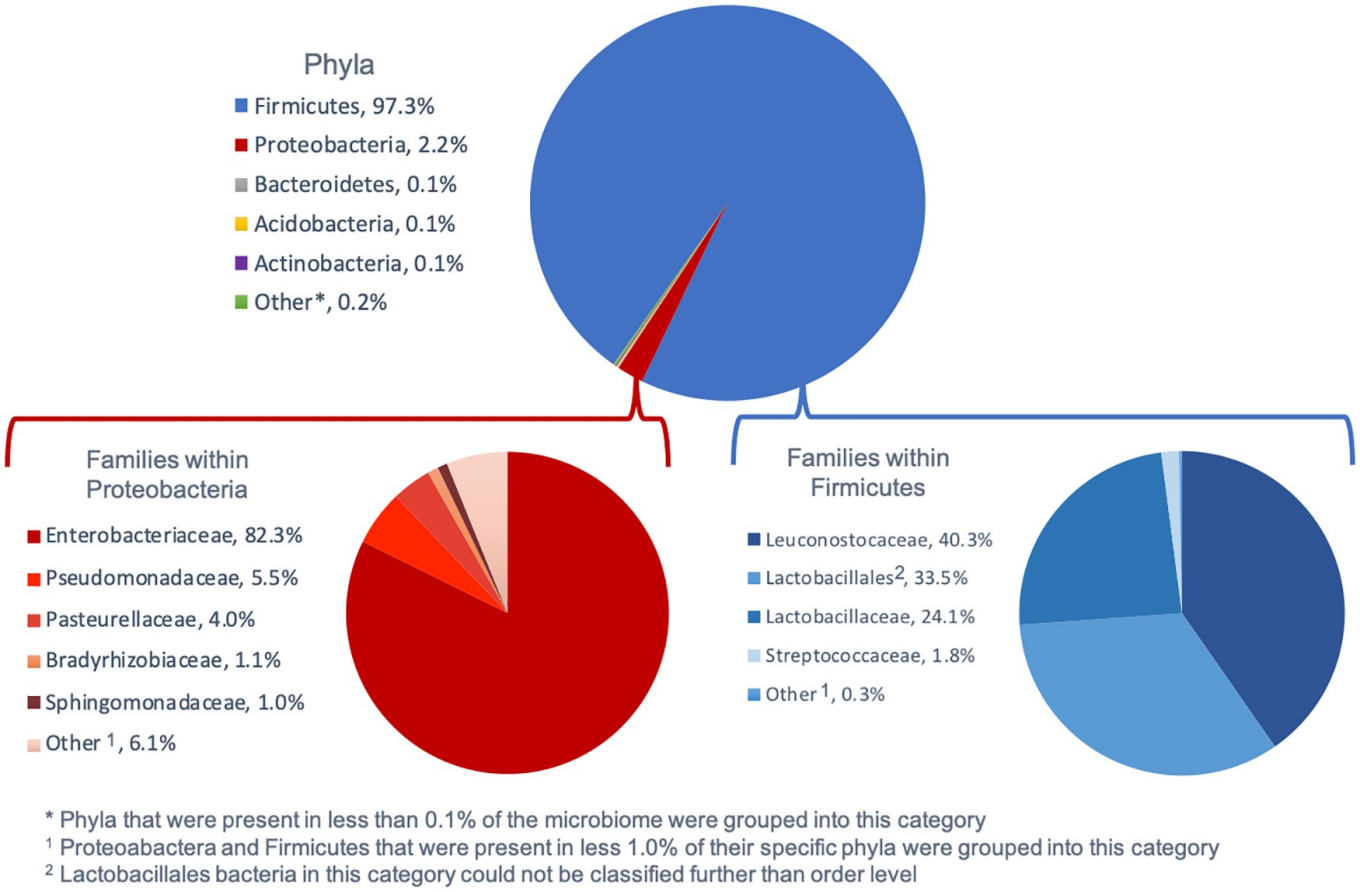
Composition of fresh ground beef microbiome across all antimicrobial treatments and days of storage.

Firmicutes was the dominate phylum of bacteria across all storage days and antimicrobial interventions, comprising 70 to 99.9% (with a mean of 97.3%) of bacterial relative abundance. Bacterial taxa in the order *Lactobacillales* (LAB) were dominant within the Firmicutes phylum and included families such as *Leuconostocaceae*, and *Lactobacillaceae* (Fig 2). The high level of LAB was not unexpected due to the use of packaging with low oxygen, and in conjunction with CO_2_, which promotes LAB growth [32]. Anaerobically packaged ground beef has been previously reported to be dominated by LAB in culture studies [6] and LAB isolation followed by 16S rRNA characterization [20].

Proteobacteria was the second most common phylum represented in the beef microbiome (Fig 2). Within Proteobacteria, the family *Enterobacteriaceae* accounted for the greatest portion of the phylum followed by *Pseudomonadaceae*. The *Enterobacteriaceae* family (which made up 1.75% of the total microbiome) encompasses a number of pathogens of interest in meat production, such as *Salmonella* spp., *E*. *coli* O157:H7, and non-O157 Shiga toxin producing *E*. *coli* (STEC); thus, *Enterobacteriaceae* is commonly used as an indicator organism for potential presence of pathogens. The rationale behind an indicator organism is that if there is a reduction of the family of bacteria, the pathogenic sub-population also will decline in magnitude accordingly [33]; though some studies have shown only limited direct relationships between indicators and pathogens [34]. The low levels of *Pseudomonadaceae* and *Streptococcaceae* found in the samples were not surprising as these bacterial families have previously been associated with spoilage of meat stored under refrigeration conditions [6, 35] (though in the case of *Pseudomonadaceae* it has been prominently associated with aerobic packaging conditions [36]).

### In addition to dominant bacteria, rare taxa were identified

While LAB and *Enterobacteriaceae* were found to comprise the majority of the bacterial community, three genera were also found in more than half of the samples: *Geobacillus* (mean = 0.07%; range 0.00 to 0.70%), *Thermus* (mean = 0.04%; range 0.00 to 0.32%), and *Sporosarcina* (mean = 0.06%; range 0.00 to 0.19%). Within extraction blanks, *Sporosarcina* had one hit across all blanks while the other two genera did not have any reads assigned to them; indicating these genera are likely the result of a biological presence, not laboratory or reagent contamination. While *Geobacillus* is an aerobic thermophile, it has been isolated from many cooler environments, such as cool soils and ocean water; which has been attributed to the longevity and mobility of this genus in the atmosphere [37]. Recently, *Geobacillus stearothermophilus* was isolated from imported frozen beef meat collected in Cairo, Egypt [38]. The genus *Thermus* is anaerobic and heat resistant, and has been found in many environments including canned meat, soil, animal feces and dust [39]. Finally, *Sporosarcina*, are gram positive strict aerobes [40] that are considered important spore-formers in meat systems [41] and have been found on meat contact surfaces in Pakistan [42]. An interesting characteristic these genera share is their characterization as extremophiles or spore formers.

### Antimicrobial intervention and sampling day independently acted on the microbiome

Before being evaluated individually, the interaction between the antimicrobial treatment applied to the beef trim and ground beef storage day was considered. The antimicrobial treatment by sampling day interaction did not affect (*P* = 0.336) overall community composition. Likewise, alpha diversity was not impacted (*P* = 0.703) by an interaction between antimicrobial treatment and sampling day.

### Antimicrobial treatment alters the microbiome and lowered *Enterobacteriaceae*

**Fig 3:**
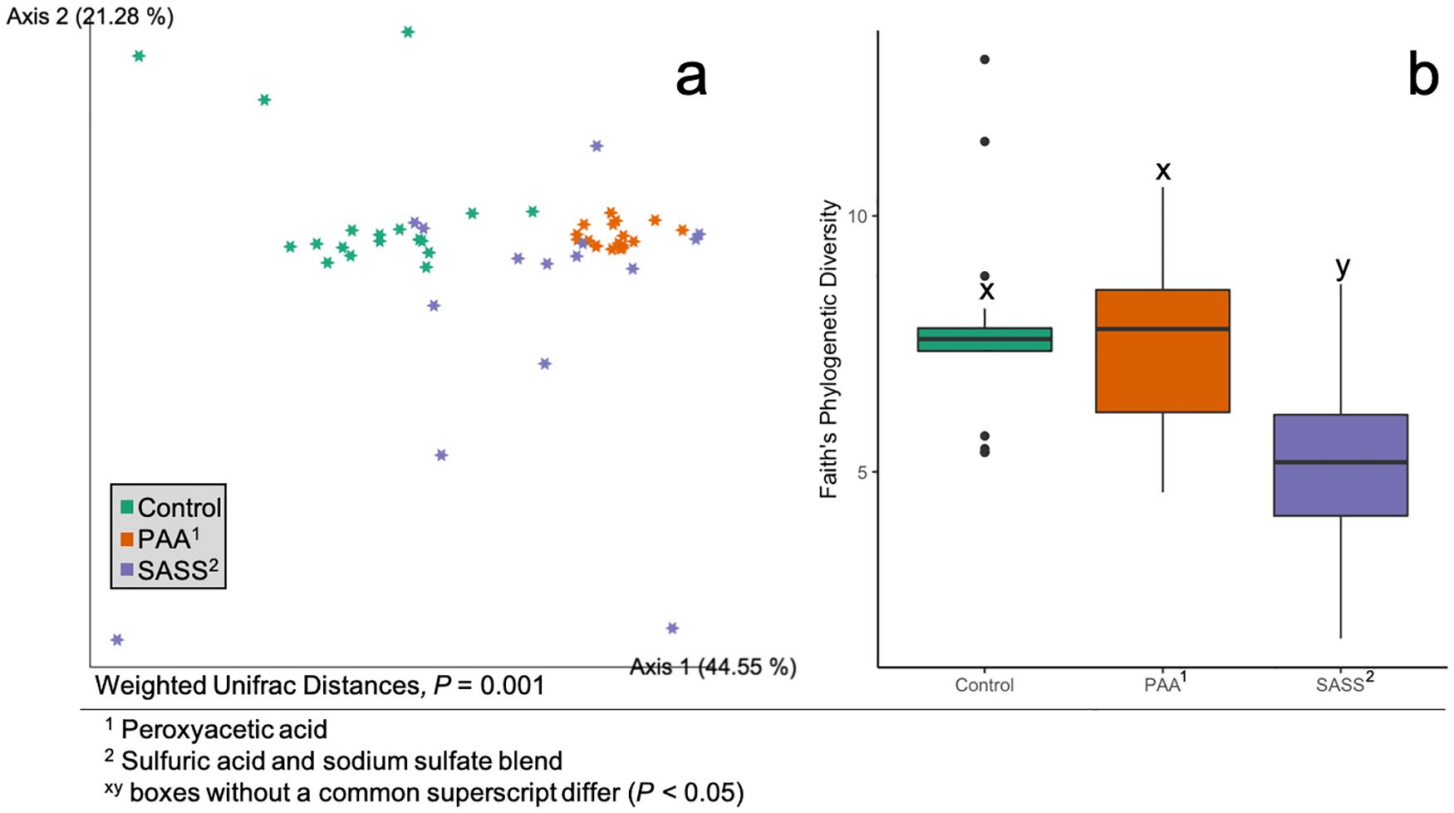
Ground beef treated with different antimicrobial treatments had different alpha and beta diversity. Effect of antimicrobial treatment of beef trimmings on ground beef microbiomes across dark storage and retail display days. (a) Total bacterial community differences between different treatments as measured by weighted Unifrac Distances were different (*P* = 0.001) and (b) alpha diversity (*P* < 0.001), as measured by Faith’s phylogenic diversity.

**Fig 4:**
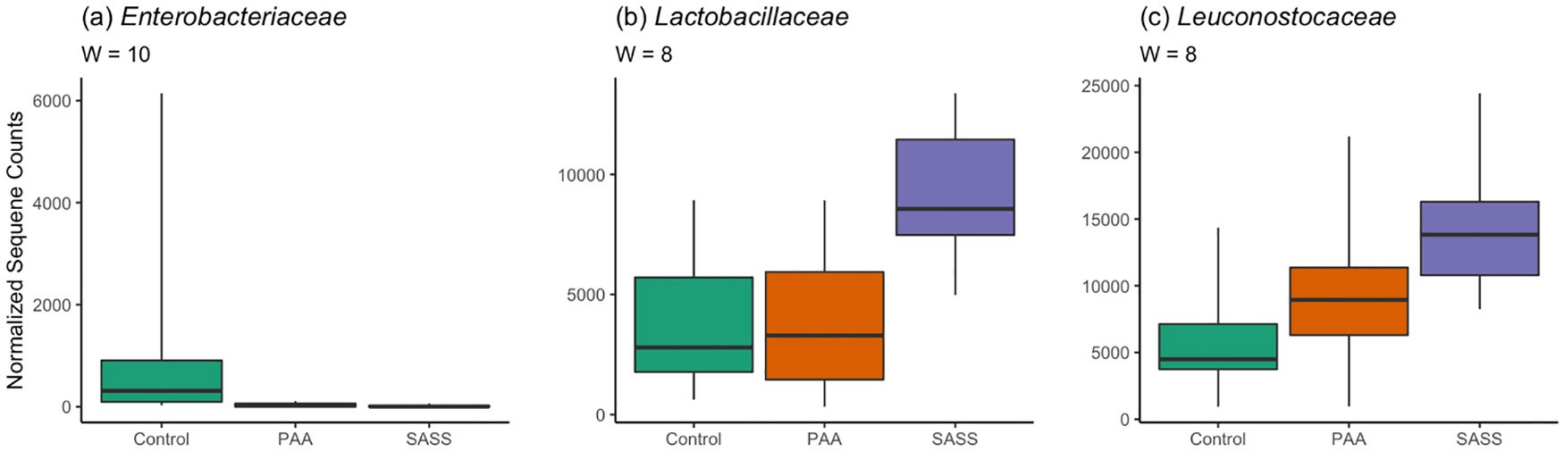
Three bacterial families differed between antimicrobial treatments: *Enterobacteriaceae*, *Lactobacillaceae*, *and Leuconostocaceae*. When analysis of composition of microbiomes (ANCOM) was conducted at the family level, 11 families were compared. Three of these families were considered significantly different: (a) *Enterobacteriaceae*, (b) *Lactobacillaceae*, and (c) *Leuconostocaceae*. The *W* statistic represents the number of pairwise comparisons that were considered significantly different. In this study, 11 families were identified across all samples.

Treatment of beef trimmings with peroxyacetic acid (PAA) or the sulfuric acid and sodium sulfate blend (SASS) prior to grinding, altered many aspects of the beef microbiome. When beta diversity was compared, antimicrobial treatments differed (*P* = 0.001, Fig 3a) from each other. This was also true in the case of alpha diversity (*P* < 0.001, Fig 3b) which was found to be lower in the SASS samples when compared to the control and PAA samples. Both chemical interventions appeared to cluster together and alter beta diversity in the same way, though SASS had an impact (*P* < 0.001) on alpha diversity while PAA did not. Three families of bacteria were found to differ (*P* < 0.05) in relative abundance across treatments: *Enterobacteriaceae*, *Lactobacillaceae*, and *Leuconostocaceae* (Fig 4). The average occurrence of *Enterobacteriaceae* was 4.7% (range 0.1 to 25.1%) in untreated control samples as compared to the SSAS (average = 0.1%, range 0.0 to 0.4%) and PAA (average = 0.2%, range 0.0 to 0.7%). The higher percentage associated with *Enterobacteriaceae* in the ground beef not treated with a chemical intervention suggests the chemical treatments are appropriate for decreasing *Enterobacteriaceae* on ground beef product. *Enterobacteriaceae* reduction as a result of interventions has previously been demonstrated throughout the beef slaughter process [43]. In previous culture-independent ground beef work [20], *Leuconostoc* spp., from the family *Leuconostocaceae* was found to be the dominant LAB organism. Interestingly, in the current study, *Leuconostocaceae* was found in a lower abundance in samples without chemical intervention (average = 22.7%, range 4.3 to 41.0%) when compared to the SASS (average = 48.8%, range 34.6 to 65.2%) and PAA (average = 46.9%, range 7.7 to 88.2%) treatments. Past antimicrobial intervention work has demonstrated that gram negative bacteria are typically more sensitive than gram positive bacteria when treated with an antimicrobial, such as a weak organic acid [44], due to differences in cell wall composition. So, while it was not a surprise to see such a high relative abundance of LAB (because they are gram positive), different LAB composition found within different chemical intervention samples may indicate that some families of LAB are more sensitive to specific chemical intervention than others. Another factor to consider, specific to the relative abundance of *Lactobacillaceae* between SASS and PAA treated ground beef, is that these differences could stem from differing pH acidities of SASS and PAA (SASS had a lower pH) that would in turn have altered the ground beef pH and LAB populations.

While a direct comparison between conventional culture methods and amplicon methods was not the intention of this study, these data provide some insight into future applications of the amplicon methodology in answering meat science questions. Identification beyond the family or genus level of bacteria was not the intention or possible with the molecular methods employed here. While other molecular methods (such as shotgun metagenomics or quantitative PCR) could provide a higher level of phylogenetic clarity, these data were intended to provide an overview of the ecological shift due to treatments—which has been demonstrated by the observed shifts in *Enterobacteriaceae*, *Lactobacillaceae*, and *Leuconostocaceae* populations. If the goal was to measure the reduction of specific pathogens without the wider context of other shifts in the microbiome, culturing on selective media or other molecular methods would have been more appropriate.

### Alpha diversity decreased over time

**Fig 5:**
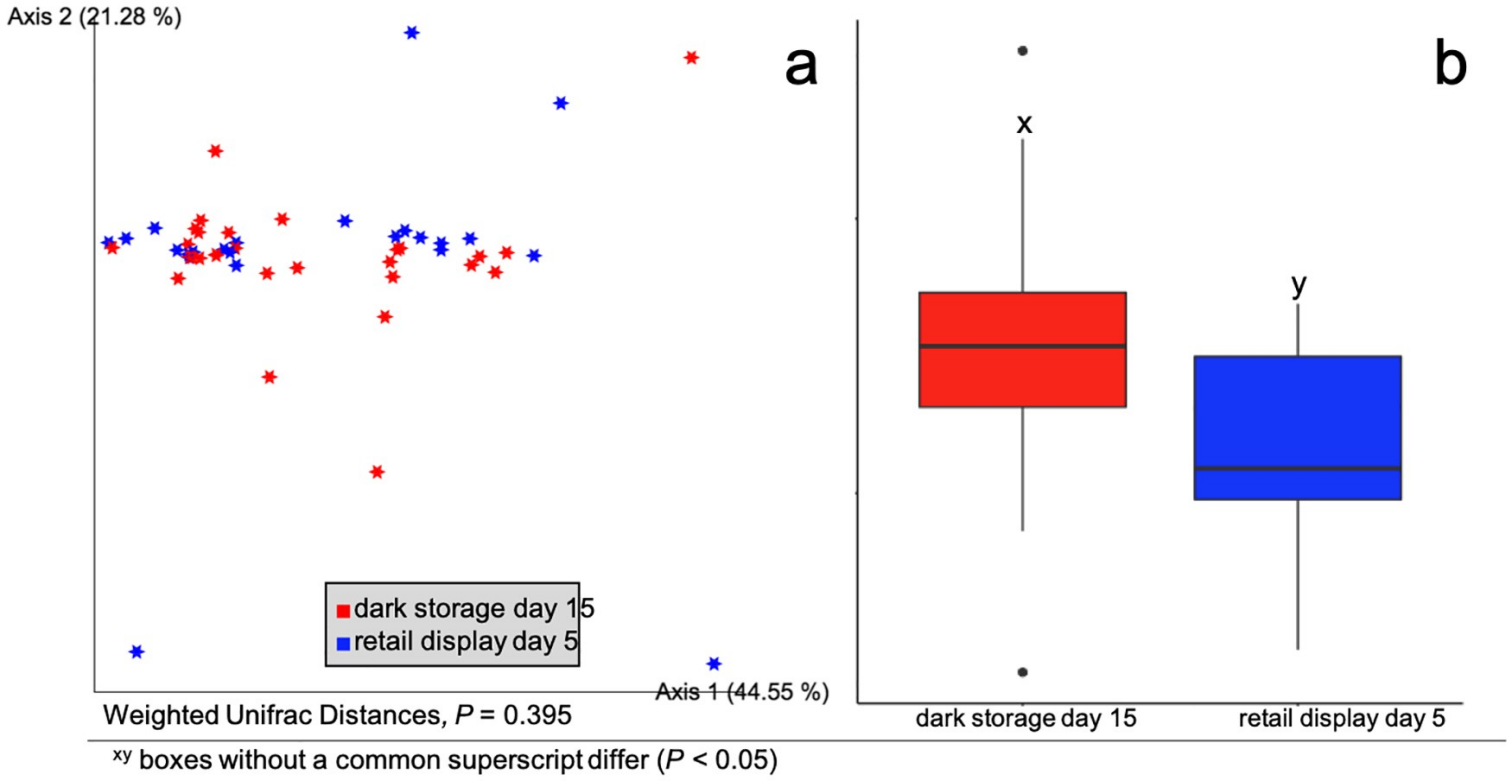
Between days of experiment, beta diversity was not different but alpha diversity did differ. Differences between storage days on ground beef microbiomes across beef trim chemical interventions. (a) Total bacterial community differences between days, as measured by weighted Unifrac Distances, were not different (*P* = 0.395) but (b) alpha diversity, as measured by Faith’s phylogenic diversity, was different (*P* = 0.001).

While beta diversity did not differ (*P* = 0.395) between day-15 of dark storage and day-5 of retail display, alpha diversity did differ (*P =* 0.001; Fig 5). Day-15 of dark storage had an average Faith’s phylogenetic diversity (FPD) of 7.57 (95% C.I. 6.93 to 8.20) while day-5 of retail display had an average FPD of 5.80 (95% C.I. 5.10 to 6.51). This finding shows a decrease in diversity paired with an increase in bacterial growth over the two time points. In other words, although biomass of bacteria increased over time, the community included fewer types. The two storage days had different (*P* = 0.04) average numbers of exact sequence variants (ESV) present; day-15 of dark storage had, on average, 21 ESVs present (95% C. I. 18 to 24), while day-5 of retail display had 16 ESVs present (95% C. I. 12 to 19). This could be due to LAB out competing other bacterial taxa over storage time. An increase in the alpha diversity of LAB has been associated with MAP packaging when compared to non-MAP, though not over duration of storage [20]. At the same time, Brooks et al. [30] observed an increase in LAB over retail display while Lavieri and Williams [31] observed the same increase in ground beef in storage. Though there was a numeric increase in relative abundance of LAB over time (1.3% on the dark storage day versus 2.3% on the retail display day), this difference was not significant (*P* > 0.05), demonstrating one of the challenges associated with amplicon sequencing data. Specifically, relative abundance is not absolute abundance within a community; if the LAB were present in 90% of a sample but the entire community was 2 logs lower than a comparison, we may not see a log fold difference. However, 16S rRNA gene sequencing does allow a level of description of the community not possible with traditional culture techniques, making the two approaches highly complementary.

### Conclusions

These data allowed for the characterization of the fresh ground beef microbiome and effects of common practices of chemical interventions and packaging on the product. Results showed that Firmicutes, mainly *Lactobacillales* (a known spoilage organism in ground beef), dominated the microbiome across all treatments while several rare genera of extremophiles or spore formers were characterized that are not typically associated with ground beef spoilage. Furthermore, *Enterobacteriaceae* was reduced as a result of antimicrobial treatment, which is consistent with culture data. One challenge of this study was the low biomass during the early days of dark storage. As a result, many of the samples on the day of grinding and in the early days of dark storage did not sequence at a sufficient depth to be included in the analysis. This result was supported by APC, which were also lower in early days of dark storage when compared to sample days later in the study. Overall, these data provide a different look at common shelf life and food safety practices in ground beef, allowing for both confirmation of dominant phyla and descriptions of lesser reported bacteria for a higher level of clarity into the changes in the microbiome, not afforded to us by culture-based methods alone.

## Supporting information

S1 Fig Unweighted UniFrac showed treatment but not day differences. Total bacterial community differences between different days (A) and treatments (B) as measured by unweighted Unifrac Distances. Day was not considered significant (*P* = 0.071) while treatment was (*P* = 0.009).
(TIF)

S2 Fig All sample’s rarefaction curves leveled. Rarefaction curve of all samples. The leveling off of the curve illustrates that an appropriate sampling depth was reached for the bacterial diversity of the community sampled.
(TIF)

S1 Table Antioxidant inclusion information. Details on antioxidant inclusion for the 52 samples included in the analysis.
(PDF)
