# Supplementary figures and images for: Ground beef microbiome changes with antimicrobial decontamination interventions and product storage
Source: PLoS One. 2019 Jun 5;14(6):e0217947. doi: 10.1371/journal.pone.0217947 (PMC6550395; doi:10.1371/journal.pone.0217947)

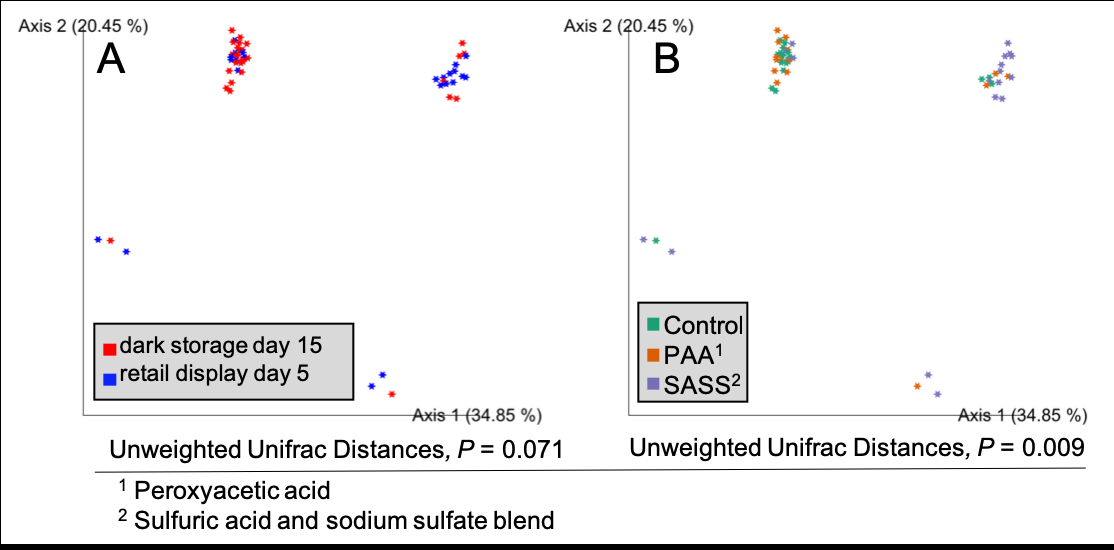

Supplement: S1 Fig — Total bacterial community differences between different days (A) and treatments (B) as measured by unweighted Unifrac Distances. Day was not considered significant (P = 0.071) while treatment was (P = 0.009). (TIF) [file pone.0217947.s001.tif]

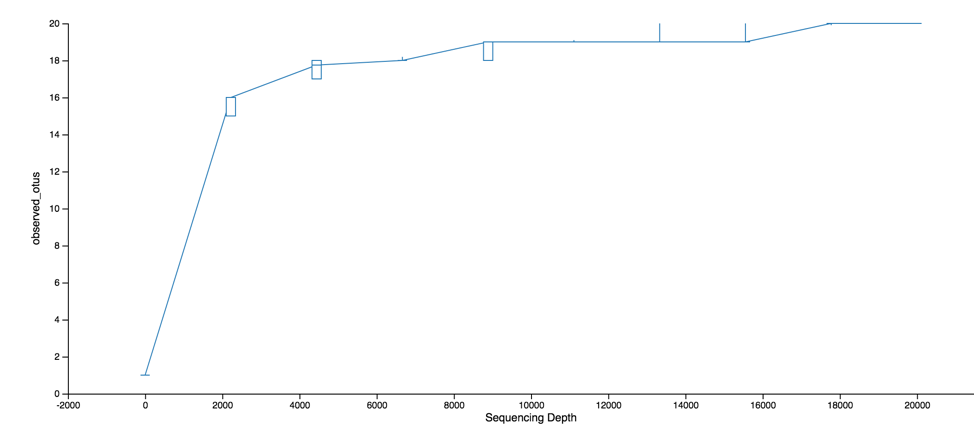

Supplement: S2 Fig — Rarefaction curve of all samples. The leveling off of the curve illustrates that an appropriate sampling depth was reached for the bacterial diversity of the community sampled. (TIF) [file pone.0217947.s002.tif]
