# Supplementary material for: Ground beef microbiome changes with antimicrobial decontamination interventions and product storage
Source: PLoS One. 2019 Jun 5;14(6):e0217947. doi: 10.1371/journal.pone.0217947 (PMC6550395; doi:10.1371/journal.pone.0217947)
Supplement: S1 Table — Details on antioxidant inclusion for the 52 samples included in the analysis. (PDF) [file pone.0217947.s003.pdf]

Table S1: Antioxidant inclusion information. Details on antioxidant inclusion for the 52 samples included in the analysis.

Storage Day	Antioxidant Inclusion	Antimicrobial Treatment Applied to Beef Trim			
		SASS[1]	None	PAA[2]	Total
Day-15 of dark storage	Yes	5	5	5	15
	No	6	5	3	14
Day-5 of retail display[3]	Yes	4	3	4	11
	No	4	5	3	12
**Total**		**19**	**18**	**15**	**52**

[1] Sulfuric acid and sodium sulfate blend

[2] Peroxyacetic acid

[3] Retail display followed 21 days of dark storage
